# Clinical Results and Safety of Intracardiac Echocardiography Guidance for Combined Catheter Ablation and Left Atrial Appendage Occlusion

**DOI:** 10.31083/j.rcm2506192

**Published:** 2024-05-27

**Authors:** Qian Liu, Ling You, Jing Yang, Yan Zhang, Jinglan Wu, Hongning Yin, Yanan Zhang, Ruiqin Xie

**Affiliations:** ^1^Department of Cardiology, The Second Hospital of Hebei Medical University, 050000 Shijiazhuang, Hebei, China; ^2^Department of Cardiac Ultrasound, The Second Hospital of Hebei Medical University, 050000 Shijiazhuang, Hebei, China

**Keywords:** atrial fibrillation, intracardiac echocardiography, left atrial appendage occlusion, radiofrequency ablation, transesophageal echocardiography

## Abstract

**Background:**

The goal of this study was to compare the procedural safety and long-term outcome associated with a combined catheter ablation and left atrial appendage occlusion (LAAO) procedure utilizing intracardiac echocardiography (ICE) guidance versus transesophageal echocardiography (TEE) guidance. The study focuses on implementing LAmbre and Watchman devices in patients diagnosed with nonvalvular atrial fibrillation (AF).

**Methods:**

A total of 363 patients diagnosed with nonvalvular AF and who underwent a combined procedure were prospectively enrolled between November 2017 and May 2022. Following 1:1 propensity score matching, the TEE group (n = 132) and ICE group (n = 132) were systematically compared in terms of the combined procedure, imaging parameters, events related to the procedure, and subsequent outcomes during follow-up, including mortality, stroke, bleeding, device-related thrombus (DRT), and peri-device leaks (PDLs).

**Results:**

The ICE group exhibited a significant reduction in total procedural duration (153.71 ± 31.71 vs. 174.74 ± 18.79 min), fluoroscopy radiation dosage (207.24 ± 108.39 vs. 268.61 ± 122.88 mGy), left atrial appendage occlusion procedure time (34.69 ± 10.91 vs. 51.46 ± 15.84 min), and contrast agent exposure (108.71 ± 37.59 vs. 158.41 ± 45.00 mL) compared to the TEE group. Angiography and ICE demonstrated a substantial correlation between the left atrial appendage (LAA) orifice and landing zone/LAA ostium (Pearson’s correlation coefficient r = 0.808 and 0.536/0.697, two-tailed *p* < 0.001). No occurrences of device-related embolism, thromboembolism, significant bleeding, or unexpected fatalities were observed in either group. Comparable rates of all-cause death (0.76% vs. 0.76%), stroke or transient ischemic attack (2.27% vs. 1.52%), severe bleeding (1.52% vs. 0.76%), PDL (23.81% vs. 24.62%), and DRT (1.52% vs. 1.52%) were noted after an average follow-up of 18.46 ± 7.70 months in both groups, with no discernible differences. Multivariate logistic regression analysis identified a correlation between LAA velocity and the risk of PDL.

**Conclusions:**

The effectiveness and safety of ICE-guided combined treatment were demonstrated to be comparable to TEE guidance, accompanied by the additional advantages of decreased procedure time and fluoroscopy radiation exposure.

**Clinical Trial Registration:**

NCT04391504, https://register.clinicaltrials.gov.

## 1. Introduction

Individuals with atrial fibrillation (AF), the predominant type of arrhythmia, face a significant risk of ischemic stroke [1]. As AF radiofrequency ablation alone does not mitigate the risk of stroke [2], it is recommended that individuals at a heightened risk of stroke continue oral anticoagulant therapy post-ablation [3]. A common approach for symptom alleviation and reducing thromboembolic risk in such patients involves atrial fibrillation catheter ablation (CA) coupled with a left atrial appendage occlusion (LAAO) procedure [4, 5]. Presently, conventional transesophageal echocardiography (TEE) is a crucial imaging modality for this combined procedure, despite associated risks such as esophageal injury [6], particularly in cases where left atrial posterior wall ablation precedes occlusion. Intracardiac echocardiography (ICE) has become increasingly popular in structural cardiology and electrophysiology in recent years due to its many benefits, including high-quality imaging, flexible probes, and no requirement for general anesthesia [7]. Since Mráz *et al*. [8] first applied ICE to LAAO in 2007, subsequent reports have consistently affirmed its efficacy and safety in the context of LAAO [8, 9, 10, 11]. However, the clinical outcomes of ICE-guided and TEE-guided combined procedures employing the LAmbre and Watchman devices over the long term have not yet been documented. This study aimed to evaluate the similarities and differences between ICE-guided and TEE-guided combined procedures using two commonly used occluders regarding clinical outcome and safety.

## 2. Methods

### 2.1 Research Participants

Between November 2017 and May 2022, a total of 363 patients undergoing a combined procedure were prospectively enrolled at a single center. Factors such as the patient’s overall health (TEE tolerance, renal function), personal willingness, and economic situation determined whether the patients were assigned to the ICE or TEE groups. The Second Hospital of Hebei Medical University’s ethics committee accepted the study protocol (No. 2020-C037), and all patients provided written informed consent. The electronic medical record system was mined for information on the baseline characteristics of patients. The current guidelines and recommendations for CA and LAAO include (1) CHA2DS2-VASc (Congestive heart failure, Hypertension, Age ≥75 years, Diabetes mellitus, prior Stroke or transient ischemic attack or thromboembolism, Vascular disease, Age 65-74 years, Sex category (female)) score of ≥2 points and HAS-BLED (Hypertension, Abnormal renal/liver function, Stroke, Bleeding history or predisposition, Labile INR, Elderly (>65 years), Drugs/alcohol concomitantly) score of ≥3 points; (2) contraindications to oral anticoagulants; (3) a history of stroke or thromboembolism during anticoagulant treatment; (4) an inclination toward opting for the catheter ablation combined with LAAO procedure, despite having received ample information. Patients were not eligible if they (1) had a left atrial or left atrial appendage (LAA) thrombus confirmed by preprocedural TEE, (2) had a history of valvular heart disease or artificial heart valve replacement, (3) were pregnant, (4) had uncontrolled hyperthyroidism, or (5) had a history of severe liver or kidney diseases, malignant tumors, or hematological diseases.

### 2.2 Combined LAAO and CA Procedure

The justification of the combined procedure is based on numerous shared procedural methods, such as femoral venous catheterization and trans-septal puncture. Preceding the intervention, both TEE and cardiac computed tomography angiography were conducted within 1–2 days. These assessments aimed to evaluate the morphology and adjacent structures and exclude the presence of an LAA thrombus. This evaluation was performed after thoroughly reviewing the patient’s medical history, demographics, and laboratory test results. During the combined procedure, AF ablation took precedence over LAAO, and both interventions were performed on conscious patients under sedation using a low dose of fentanyl (0.1 mg/h). The protocol included the intravenous administration of heparin, targeting an activated clotting time in the range of 250 to 350 seconds.

The present study incorporated the utilization of two distinct LAAO devices: the Watchman (Boston Scientific, Marlborough, MA, USA) and the LAmbre (Lifetech Scientific, Shenzhen, China). The Watchman, designed as a single-seal device resembling an umbrella, seals off the LAA. Unlike the Watchman, the LAmbre closing system received Conformité Européenne (CE) mark clearance on June 15, 2016. This system comprises nitinol-based occluders featuring a fabric-enriched cover, an umbrella with a short central waist, and a single attachment hub. The selection of the device type was conducted by the attending physicians, who considered the anatomical characteristics of the LAA.

**Fig. 1.**
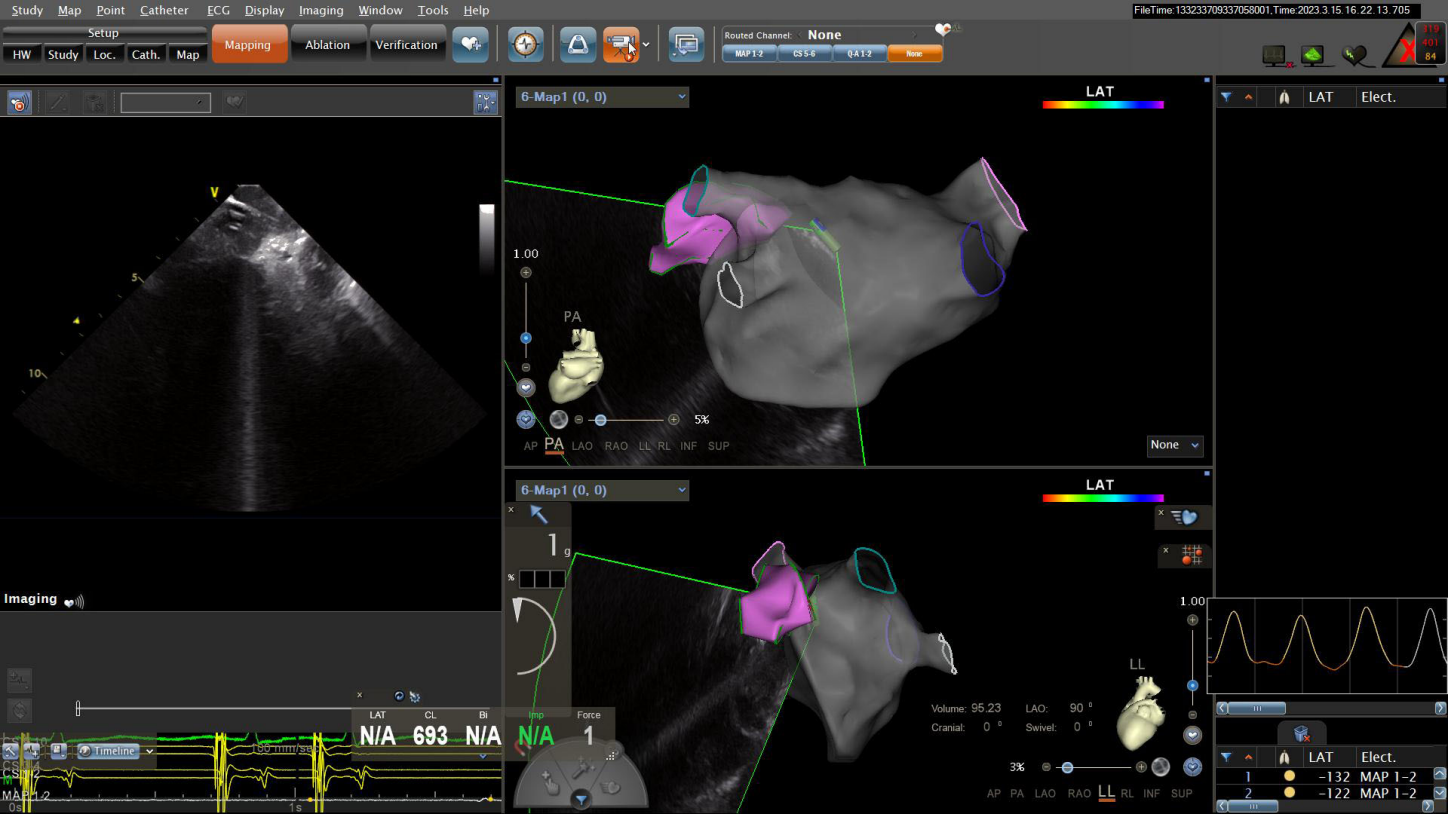
**Utilizing intracardiac echocardiography to model the left atrial and left atrial appendage geometry map.** LAT, local activation time; PA, posteroanterior; LL, left lateral; N/A, not available.

In the ICE group, the ICE catheter (AcuNav; Johnson & Johnson, New Brunswick, NJ, USA) was introduced into the heart via the right femoral vein. Employing the CARTOSOUND module and three-dimensional mapping technology (CARTO 3; Biosense Webster Inc., Diamond Bar, CA, USA), the geometry map of the left atrium and LAA was constructed. Subsequently, this map was fused with the preprocedural cardiac computed tomography angiography image, as illustrated in Fig. 1.

In the TEE group, fluoroscopic guidance was employed for dual punctures of the atrial septum. Conversely, in the ICE group, the puncture was carried out under the guidance of ICE. Following the established methodology outlined in a prior study [12], radiofrequency ablation was executed utilizing the CARTO 3 electrophysiological mapping system. Pulmonary vein isolation was assessed using a circular mapping cathete. The anterior wall was ablated at 45 W with an ablation index of 450, whereas the posterior wall was ablated at 40 W. The ablation procedure was deemed successful when the pulmonary vein potential vanished and the ablation circles or lines were effectively blocked in both directions.

**Fig. 2.**
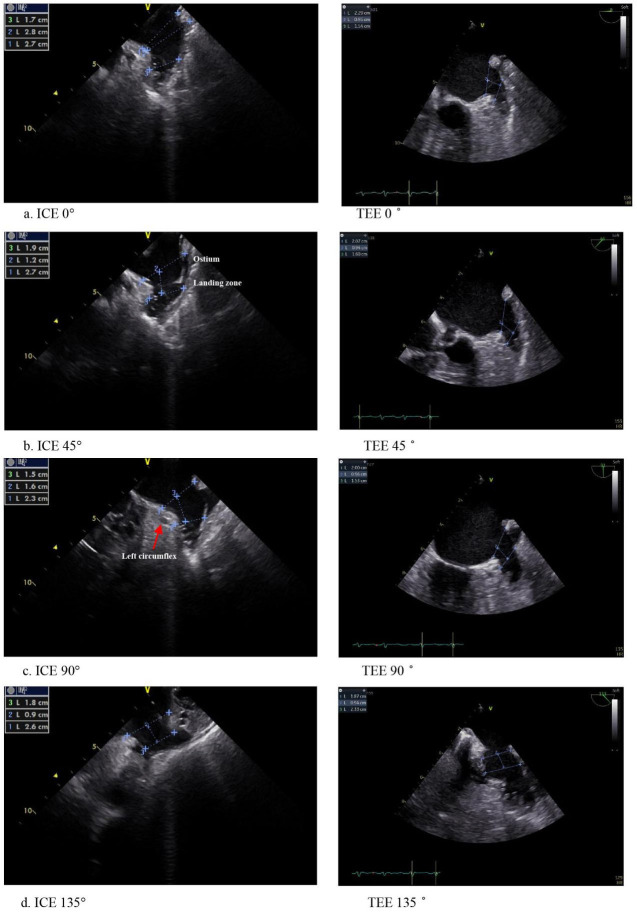
**ICE compared to TEE in the same patient at 0°, 45°, 90°, and 135°.** The 0° view: The LAA was scanned horizontally from posterior to anterior, which was positioned at the top of the left inferior pulmonary vein. The 45° view: The LAA was taken at 45° from posteroinferior to anterosuperior by inserting the ICE probe into the left superior pulmonary vein ostium. The 90° view: Views of the LAA from right to left, similar to the 90° TEE view, were obtained by withdrawing the ICE probe from the left superior pulmonary vein to the center of the left atrium and rotating the sector to the mitral valve. This was the optimal vantage point from which to study the link between the device and the mitral annulus. The 135° view: the ICE probe was positioned in the center of the left atrium, with the sector pointing toward the back of the right pulmonary vein and not far from the mitral annulus. ICE, intracardiac echocardiography; TEE, transesophageal echocardiography; LAA, left atrial appendage.

ICE-Guided LAAO. Using the pre-established three-dimensional map of the left atrium, the ICE probe was introduced through a puncture in the atrial septum. The probe was positioned near the left inferior pulmonary vein, the left superior pulmonary vein, the top of the left atrium, and the mitral annulus. This positioning aimed to acquire images akin to those obtained through the TEE echo window approach at 0°, 45°, 90°, and 135° immediately following the ablation procedure. The acronym “XR-Star” stands for “X-ray reduction LAAO workflow with the simulation of four TEE angles guided by the SOUNDSTAR catheter”, which is the name given to this imaging method (Fig. 2). TEE images obtained at various angles have distinct features [13]. ICE was used to scan the long axis of the LAA at various angles to determine its maximum cross-section.

Following a meticulous examination of the fluoroscopy and ICE data, a device with the appropriate right occluder and size was selected and positioned within the LAA. Subsequently, the umbrella was deployed under continuous ICE monitoring. A tug test was performed, and assessments of the peri-device leakage and device stability were conducted at four different angles (0°, 45°, 90°, and 135°). Upon achieving stability in the tug test, with peri-device leaks (PDL) measuring less than 3 mm, in accordance with either COST (left circumflex, open, sealing, tug test) or PASS (position, anchor, size, seal) criteria, the device was subsequently disconnected.

TEE-guided LAAO. Following CA for AF, LAA angiography and preprocedural TEE measurements were used to select the appropriate occluder and size. Under fluoroscopic guidance, the delivery catheter and device were maneuvered to the intended location. Subsequently, the placement of the device and the amount of residual peri-device flow were validated using TEE, which was performed while the patient was under local anesthetic.

### 2.3 Anti-Arrhythmic Medications and Anti-Thrombotic Therapy

Patients with persistent/long-standing persistent AF are typically administered amiodarone via oral and intravenous transfusion before undergoing a combined procedure. However, patients with paroxysmal AF are not administered antiarrhythmic medications. Three months after the combined procedure, all patients received oral amiodarone. Patients were prescribed rivaroxaban or dabigatran as anticoagulants during the initial 3 months following the combined procedure. At 3 months, TEE was scheduled to detect any PDL and device-related thrombus (DRT). Subsequently, patients were transitioned to a regimen of aspirin (100 mg/day) and clopidogrel (75 mg/day) until the six-month mark. If no PDL was detected, or if the PDL measured less than 5 mm and no DRT was observed, patients were shifted to aspirin alone until the twelve-month mark.

Ultrasound equipment was used to measure and image the LAA in the ICE and TEE groups. A General Electric Vivid I system (General Electric, Boston, MA, USA) was employed in the ICE group, whereas the TEE group utilized an iE33 Color Ultrasound system (Philips, Amsterdam, Netherlands). For the Watchman device, the widest ostium (the length from the circumflex artery to 10–20 mm within the pulmonary venous ridge) and the LAA depth (the length from the LAA ostium to the farthest end of the selected distal lobe) were measured. On the other hand, for the LAmbre device, measurements comprised the widest LAA orifice (the line connecting from the pulmonary vein ridge superiorly to the inferior junction of the left atrial (LA)/LAA at the circumflex artery) and the widest landing zone, determined as the maximum diameter obtained at 10 mm in the ostium.

During the combined procedure, the total procedural time, fluoroscopy radiation dose (from femoral vein puncture to device release), LAAO procedural time, fluoroscopy radiation dose (from second atrial septum puncture to LAAO completion), and contrast agent dose were recorded. Outpatient clinic visits or telephone consultations were planned for 3, 6, 12, and 24 months post-operation. TEE was conducted three months post-procedure to detect device-related thrombus development, device displacement, and PDL. During each subsequent follow-up visit, a 12-lead electrocardiogram and 24-hour Holter monitoring were advised to assess the potential recurrence of atrial tachyarrhythmia. The primary safety endpoint encompassed peri-procedural complications, specifically focusing on occurrences such as cardiac tamponade, device embolism, thromboembolism, hemorrhage, mortality, and femoral complications. The latter included subcutaneous hematoma, arteriovenous leakage, femoral vein thrombosis, and pseudoaneurysm, all assessed within the initial 7 days following the index surgery. During the follow-up period, adverse events included all-cause death and stroke. Arrhythmia recurrence was defined as any atrial tachyarrhythmia lasting more than 30 seconds after a 90-day blanking period.

### 2.4 Statistical Analysis

SPSS software version 22.0 (IBM Corp., Armonk, NY, USA) was used to analyze the data. To mitigate selection bias, patients undergoing ICE-guided and TEE-guided procedures were matched in a 1:1 ratio for age, gender, CHA2DS2-VASc score, and HAS-BLED score. Matching was accomplished within a caliper of 0.25 on the propensity score. The Kolmogorov–Smirnov test was utilized to determine if the data followed a normal distribution. The mean ± standard deviation represents continuous variables, whereas the percentage and count represent categorical variables. The paired Student’s *t*-test or the chi-squared test was used to compare differences between the two groups. Pearson’s correlation coefficient was used to examine correlations between continuous variables. A logistic regression model was utilized in the multivariable study. Cumulative event probabilities were assessed using the Kaplan–Meier and log-rank tests to compute the *p*-value. A *p*-value of <0.05 was considered statistically significant. We also estimated standardized mean differences (SMD) to investigate group bias. For continuous variables, $S\,M\,D=\frac{\bar{X_{1}}-\bar{X_{2}}}{\sqrt{\left(S_{1}^{2}+S_{2}^{2}\right)/2}}$ ($\bar{X}$, arithmetic mean; S, standard deviation); for categorical variable, $S\,M\,D=\frac{\hat{P_{1}}-\hat{P_{2}}}{\sqrt{\left[\hat{P_{1}}\,\left(1-\hat{P_{1}}\right)+\hat{P_{2}}\,\left(1-\hat{P_{2}}\right)\right]/2}}$ ($\hat{P}$, incidence rate). The balance of potential baseline confounders was assessed using standardized mean differences, with an a priori significance level of 0.20.

## 3. Results

### 3.1 Baseline Characteristics

There were 133 successful ICE procedures and 230 successful TEE procedures among the patients who underwent combined procedures. The 264 patients who were matched based on their propensity scores were split evenly between the ICE group (mean age, 62.44 ± 8.52 years; 80 men) and the TEE group (mean age, 62.98 ± 8.14 years; 76 men). In the TEE and ICE groups, the AF temporal pattern was persistent/long-standing persistent AF in 82.58% and 72.73%, respectively. The baseline characteristics of the patients are displayed in Table 1. There was no significant difference between the two groups regarding age, sex, medical history, CHA2DS2-VASc, HAS-BLED scores, left atrial diameter, LAA velocity, and LAA morphological types, and the standardized mean differences were mainly within 0.2. A total of 64 Watchman devices were implanted in the TEE group, with 66 in the ICE group, whereas 68 LAmbre devices were implanted in the TEE group and 66 in the ICE group. Device size reselection occurred in 6.82% of the patients in the ICE group and 9.85% in the TEE group, and the difference was not statistically significant (*p* = 0.37).

**Table 1.** **Comparison of demographic and procedure characteristics between TEE group and ICE groups**.

Basic characteristics			TEE (n = 132)	ICE (n = 132)	SMD	*p*-value
Age (year)			62.44 ± 8.52	62.98 ± 8.14	–0.065	0.612
Male (%)			80 (60.61)	76 (57.58)	0.0617	0.617
AF types						
	Paroxysmal AF (%)		23 (17.42)	36 (27.27)	–0.238	0.055
	Persistent/long-standing persistent AF (%)		109 (82.58)	96 (72.73)	0.238	0.055
	Hypertension (%)		88 (66.67)	83 (62.88)	0.0794	0.519
	Coronary heart disease (%)		64 (48.48)	71 (53.79)	–0.106	0.389
	Diabetes mellitus (%)		26 (19.70)	37 (28.03)	–0.196	0.112
	Heart failure (%)		98 (74.24)	85 (64.39)	0.215	0.083
	History of strokes/TIAs/SE (%)		70 (53.03)	71 (53.79)	–0.0152	0.902
	History of major bleeding (%)		4 (3.03)	5 (3.79)	–0.0419	0.734
	CHA2DS2–VAS score		4.26 ± 1.42	4.33 ± 1.44	–0.049	0.676
	HAS–BLED score		2.77 ± 0.95	2.80 ± 0.80	–0.034	0.713
Echocardiographic parameters						
	Left atrial diameter (mm)		40.90 ± 5.42	40.08 ± 5.34	0.152	0.216
	LAA velocity (cm/s)		39.02 ± 21.15	42.83 ± 22.99	–0.172	0.136
	LAA morphology types					
	Chicken wing		24 (18.18)	20 (15.15)	0.0814	0.509
		Windsock	16 (12.12)	10 (7.58)	0.153	0.215
	Cauliflower		85 (64.39)	94 (71.21)	–0.146	0.236
		Cactus	7 (5.30)	8 (6.06)	–0.0328	0.790
	Device type of final implantation					
		Watchman	64 (48.48)	66 (50.00)	–0.0304	0.806
		LAmbre	68 (51.52)	66 (50.00)	0.0304	0.806
	Device size reselection		13 (9.85)	9 (6.82)	0.110	0.373

Data are presented as n (%) or mean ± standard deviation. TEE, transesophageal echocardiography; ICE, intracardiac echocardiography; TIAs/SE, transitory ischemic attacks/stroke episodes; LAA, left atrial appendage; SMD, standardized mean differences; AF, atrial fibrillation; CHA2DS2-VASc, Congestive heart failure, Hypertension, Age ≥75 years, Diabetes mellitus, prior Stroke or transient ischemic attack or thromboembolism, Vascular disease, Age 65-74 years, Sex category (female); HAS-BLED, Hypertension, Abnormal renal/liver function, Stroke, Bleeding history or predisposition, Labile INR, Elderly (>65 years), Drugs/alcohol concomitantly.

### 3.2 Procedural Characteristics

**Fig. 3.**
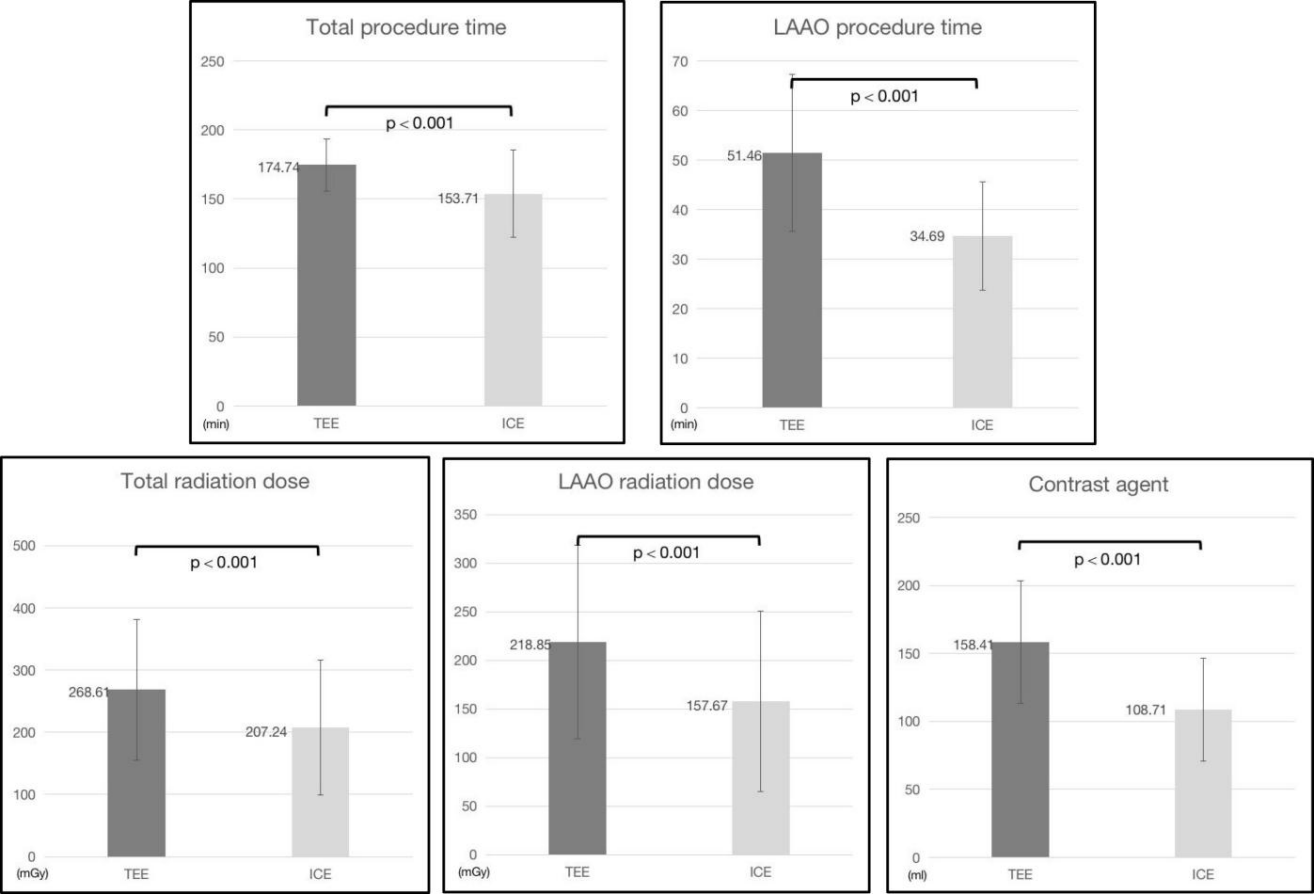
**Comparison of procedural characteristics between the TEE and ICE groups.** TEE, transesophageal echocardiography; ICE, intracardiac echocardiography; LAAO, left atrial appendage occlusion.

Fig. 3 depicts the procedural characteristics of the two groups. The mean total procedural time in the TEE and ICE groups was 174.74 ± 18.79 and 153.71 ± 31.71 minutes, respectively, and the mean subsequent LAAO procedural time was 51.46 ± 15.84 and 34.69 ± 10.91 minutes, respectively. The ICE group had a considerably shorter total procedural time, fluoroscopy radiation dosage, LAAO procedural time, and contrast agent dose than the TEE group (*p* < 0.001).

A comparative analysis was performed between preprocedural TEE and intra-procedural ICE measurements for 132 patients in the ICE group (refer to Table 2). Notably, at 90° and 135°, the ICE measurements exhibited larger dimensions than the LAA orifice/ostium TEE measurements.

**Table 2.** **LAA measurements in the ICE group by preprocedural TEE and intra-procedural ICE**.

		Preprocedural TEE	Intra-procedural ICE	*p*-value
LAmbre		N = 66	N = 66	
	Landing zone at 0° (mm)	20.60 ± 3.50	19.91 ± 4.37	0.438
	LAA orifice at 0° (mm)	24.98 ± 4.96	24.74 ± 4.20	0.823
	Landing zone at 45° (mm)	19.38 ± 3.50	19.91 ± 4.05	0.478
	LAA orifice at 45° (mm)	24.49 ± 4.50	24.79 ± 3.95	0.727
	Landing zone at 90° (mm)	19.48 ± 3.88	20.63 ± 3.79	0.126
	LAA orifice at 90° (mm)	24.07 ± 5.35	26.71 ± 3.84	0.006
	Landing zone at 135° (mm)	21.15 ± 4.90	22.70 ± 4.21	0.080
	LAA orifice at 135° (mm)	25.25 ± 4.60	27.54 ± 3.71	0.005
Watchman		N = 66	N = 66	
	LAA ostium at 0° (mm)	19.31 ± 3.32	19.85 ± 3.61	0.482
	Effective depth at 0° (mm)	25.28 ± 5.14	23.90 ± 4.70	0.209
	LAA ostium at 45° (mm)	18.33 ± 3.54	19.33 ± 3.33	0.138
	Effective depth at 45° (mm)	24.88 ± 5.06	23.79 ± 4.79	0.262
	LAA ostium at 90° (mm)	18.47 ± 4.06	19.82 ± 2.86	0.055
	Effective depth at 90° (mm)	24.75 ± 5.74	23.04 ± 4.61	0.101
	LAA ostium at 135° (mm)	19.16 ± 3.84	20.48 ± 3.53	0.073
	Effective depth at 135° (mm)	23.67 ± 4.98	22.91 ± 4.06	0.403

Data are presented as mean ± standard deviation. LAA, left atrial appendage; TEE, transesophageal echocardiography; ICE, intracardiac echocardiography.

**Fig. 4.**
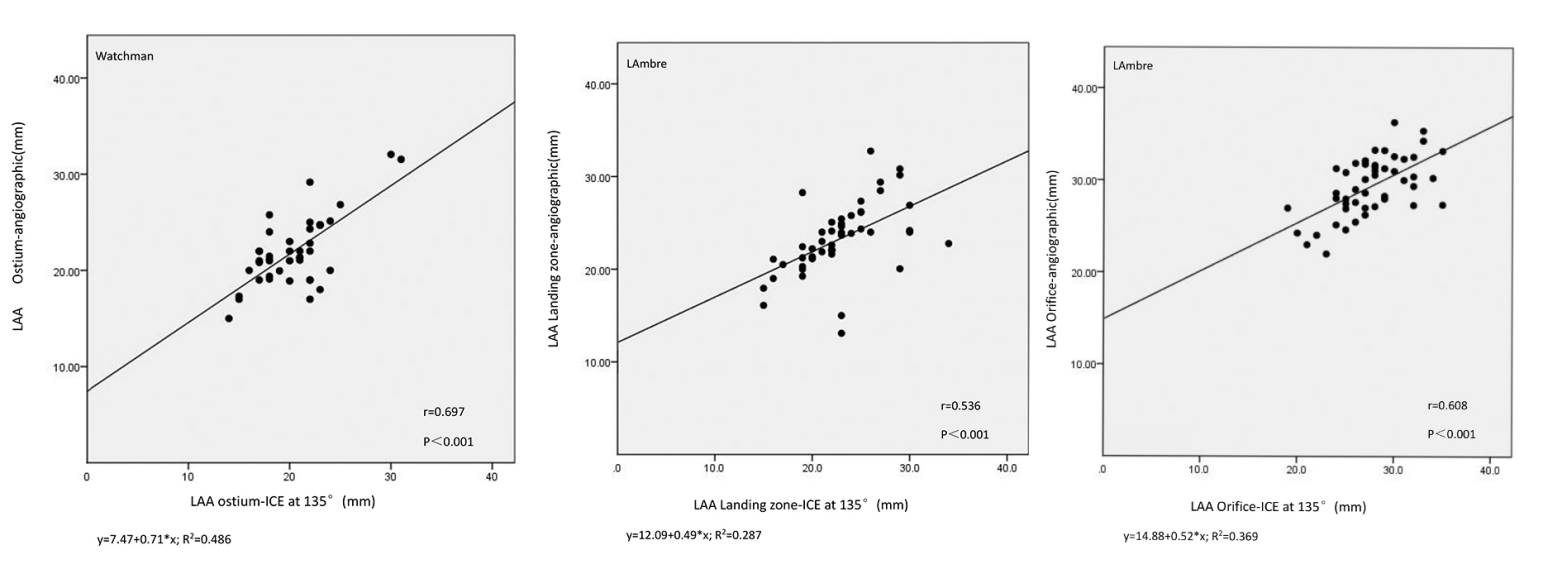
**Graph demonstrating a significant correlation between angiographic and ICE measurements of the LAA orifice and landing zone for the LAmbre device and the LAA ostium for the Watchman device.** ICE, intracardiac echocardiography; LAA, left atrial appendage.

A comparison was conducted between the LAA orifice and landing zone/LAA ostium measurements obtained by ICE at 135° and angiography in the right anterior oblique caudal view for 132 cases (Fig. 4). Specifically, when assessing the diameter of the LAA orifice and landing zone for the LAmbre device, ICE yielded values of 27.54 ± 3.71 mm and 22.70 ± 4.21 mm, respectively. In contrast, fluoroscopy produced values of 29.10 ± 3.55 mm and 22.92 ± 4.05 mm, respectively. The mean LAA ostium diameter for the Watchman device measured using ICE was 20.48 ± 3.53 mm, and the diameter measured using fluoroscopy was 21.85 ± 3.42 mm. The LAA orifice and landing zone/LAA ostium were substantially correlated using angiography and ICE (Pearson’s correlation coefficient r = 0.808 and 0.536/0.697, two-tailed *p* < 0.001).

### 3.3 Peri-Procedural Events

Table 3 depicts procedure-related events. The overall rate of major procedural problems was comparable in both groups and occluders. The incidence of cardiac tamponade was 4/132 (3.03%) in the TEE group and 3/132 (2.27%) in the ICE group (*p* = 0.702); 4/130 (3.08%) with the Watchman and 3/134 (2.24%) with the LAmbre devices (*p* = 0.968). Additionally, there was no significant difference in the incidence of femoral complications between the two groups and devices (*p* = 0.478/*p* = 0.983 vs. *p* = 0.561/*p* = 0.979 vs. *p* = 0.614/*p* = 0.636). There were no cases of device-related embolism, thromboembolism, significant bleeding, or unexpected fatalities in either group.

**Table 3.** **Procedure-related events**.

Procedure complications	Total (N = 264)	TEE (N = 132)	ICE (N = 132)	*p*-value	Watchman (N = 130)	LAmbre (N = 134)	*p*-value
Cardiac tamponade	7 (2.65)	4 (3.03)	3 (2.27)	0.702	4 (3.08)	3 (2.24)	0.968
Device-related embolism	0 (0)	0 (0)	0 (0)	>0.999	0 (0)	0 (0)	>0.999
Thromboembolism	0 (0)	0 (0)	0 (0)	>0.999	0 (0)	0 (0)	>0.999
Major bleeding	0 (0)	0 (0)	0 (0)	>0.999	0 (0)	0 (0)	>0.999
Death	0 (0)	0 (0)	0 (0)	>0.999	0 (0)	0 (0)	>0.999
Femoral complications	4 (1.52)	1 (0.76)	3 (2.27)	0.614	2 (1.54)	2 (1.49)	0.636

TEE, transesophageal echocardiography; ICE, intracardiac echocardiography.

### 3.4 Long-Term Follow-Up and Post-Procedural Imaging

Table 4 displays the findings of the follow-up examination, conducted after a mean interval of 18.46 ± 7.70 months. There was no statistically significant difference between the groups or the two occluders concerning any of the key efficacy endpoint components. Cardiovascular and unexplained fatalities occurred in 1/132 (0.76%) of the TEE group and 1/132 (0.76%) of the ICE group (*p* > 0.999); both groups employed LAmbre devices. All-cause stroke occurred in 3/132 (2.27%) of the TEE group vs. 2/132 (1.52%) of the ICE group (*p* = 0.652) and in 2/130 (1.54%) of the Watchman group vs. 3/134, (2.24%) of the LAmbre devices (*p* = 0.676). Furthermore, the bleeding events occurred with a comparable frequency (2/132, 1.52% vs. 1/132, 0.76%; *p* = 0.561 and 1/130, 0.77% vs. 2/134, 1.49%, *p* = 0.622). Transoesophageal echocardiography was performed at 3 months for 126/132 (95.45%) in the TEE group and 130/132 (98.48%) in the ICE group. There was no difference in PDL events (30/126, 28.31% vs. 32/132, 24.62%; *p* = 0.780) and DRT events (2/132, 1.52% vs. 2/132, 1.52%; *p* > 0.999) in the TEE and ICE groups. The multivariate logistic regression analysis demonstrated that LAA velocity was related to an increased risk of PDL (OR = 0.964, 95% CI: 0.944–0.984, *p* < 0.001) (**Supplementary Fig. 1**). The risk of PDL was lowered by 3.5% for every 1 cm/s increase in LAA velocity. When all the above-mentioned components of the key efficacy and safety endpoints were considered, the clinical patient benefit was comparable for both groups and devices.

**Table 4.** **Follow-up results**.

Follow-up results	Total (N = 264)	TEE (N = 132)	ICE (N = 132)	*p*-value	Watchman (N = 130)	LAmbre (N = 134)	*p*-value
Death (all-cause)	2 (0.76)	1 (0.76)	1 (0.76)	>0.999	0 (0%)	2 (1.49)	0.491
Stroke or TIA	5 (1.89)	3 (2.27)	2 (1.52)	0.652	2 (1.54)	3 (2.24)	0.676
Bleedings	3 (1.14)	2 (1.52)	1 (0.76)	0.561	1 (0.77)	2 (1.49)	0.622
Number of patients with TEE follow-up	256 (96.97)	126 (95.45)	130 (98.48)	0.281	125 (96.15)	131 (97.76)	0.687
DRT	4 (1.52)	2 (1.52)	2 (1.52)	>0.999	0 (0)	4 (2.99)	0.139
No PDL	194 (75.78)	96 (76.19)	98 (75.38)	0.780	101 (80.80)	93 (70.99)	0.067
PDL 0–3 mm	56 (21.88)	26 (20.63)	30 (23.08)	0.547	22 (17.60)	34 (25.95)	0.106
PDL 3–5 mm	6 (2.34)	4 (3.17)	2 (1.54)	0.680	2 (1.60)	4 (3.05)	0.707
PDL >5 mm	0 (0)	0 (0)	0 (0)	>0.999	0 (0)	0 (0)	>0.999

TEE, transesophageal echocardiography; ICE, intracardiac echocardiography; DRT, device-related thrombus; PDL, peri-device leaks; TIA, transitory ischemic attack.

## 4. Discussion

While the viability of combining AF radiofrequency ablation with LAAO has been recently clarified [14, 15], there are two drawbacks to the combined procedure [16, 17]: (1) the possibility of increased periprocedural risks and (2) the possibility of inaccurate device selection due to post-ablation atrial edema and stunning, leading to long-term PDL and device migration. Therefore, this study aimed to investigate the potential of ICE as a substitute for TEE, currently regarded as the gold standard, in combined procedures and to assess its long-term safety. Our findings indicate that ICE is a viable alternative to TEE in selecting and deploying occluders during the combined procedure and in the post-plugging evaluation. In addition, we showed that a combined procedure using ICE guidance with the Watchman and LAmbre occluders resulted in a shorter procedure time, a lower fluoroscopy radiation dosage, and a similar complication rate and clinical results compared with TEE guidance.

### 4.1 Procedure Time and Fluoroscopy Radiation Dose

Several recent studies have studied the time required for LAAO procedures using ICE and TEE. Streb *et al*. [18] observed that the LAAO procedure took less time when guided by ICE than by TEE (45 vs. 54 min, *p* = 0.02). According to a study by Korsholm *et al*. [19], the ICE group spent considerably less time in the catheterization laboratory than the TEE group (87 vs. 116 min, *p* < 0.01). According to research by Chu *et al*. [20], time spent on the LAAO technique did not differ significantly between the ICE and TEE groups (73.0 ± 21.4 vs. 79.0 ± 58.8 min, *p* = 0.804). When comparing patients who received ICE (n = 391) and TEE (n = 766), Velagapudi *et al*. [21] found no statistically significant differences in fluoroscopy time (mean difference, 1.84; 95% CI, 0.59–4.27; *p* = 0.14) or total procedure time (mean difference, –5.06; 95% CI, –24.6 to –14.4; *p* = 0.61). Contradictory findings were also found for several procedural parameters across studies, which we attribute in part to methodological variations, the absence of a standardized protocol, and the time-dependent nature of the learning curve associated with the process. With ICE guidance, the combined ablation and LAAO treatment required considerably less fluoroscopy radiation than the TEE guidance. The observed reduction in procedure time is primarily ascribed to several contributing factors. Firstly, using ICE proves advantageous in visualizing the internal structure of the LAA, effectively acting as a supplementary “third eye” and offering more precise guidance. This enhanced clarity facilitates formulating a well-informed occlusion strategy, thereby diminishing the need for frequent occluder changes and reducing occlusion time, particularly in cases involving complex left atrial appendages. Additionally, factors such as anesthetic induction or intubation may influence the extended procedure time in the TEE group. As proficiency using XR-Star technology advances, the capability to execute the combined ablation and LAAO procedure has significantly improved, resulting in minimal to no X-ray exposure. This achievement is attributed to the continuous reduction in fluoroscopy radiation dose and contrast consumption.

### 4.2 LAA Measurements

In accordance with prior research, measurements of the LAA obtained through ICE exhibit a strong correlation with those derived from cardiac computed tomography and TEE. Masson *et al*. [22] achieved comparable LAA measurements of the orifice and landing zone using ICE and TEE. Italiano *et al*. [23] discovered a strong correlation between the various imaging modalities when comparing LAA dimensions in patients undergoing LAAO using two- and three-dimensional TEE, cardiac computed tomography, and conventional cardiac angiography. TEE is considered preferable to ICE due to its capacity for three-dimensional imaging, a capability that was previously unavailable with ICE. As a guidance tool for LAAO, Khalili *et al*. [24] found that three-dimensional volume ICE guidance was consistent and on par with TEE. However, contrary to the measurements obtained using TEE, the ICE measurements were found to be higher in some studies [11, 25], which is consistent with our findings—intraprocedural ICE measurements of the LAA ostium were larger than preprocedural TEE measurements at 90° and 135°. In light of this revelation, two hypotheses were examined. Firstly, it was postulated that the state-of-the-art phased catheter ICE system more effectively visualizes the intricate LAA structure than TEE. This is attributed to the ICE system’s superior tissue penetration, flexible catheter head, and Doppler imaging capabilities. Secondly, the observed discrepancies in measurements were considered to potentially arise from variations in left atrial pressure and LAA function, particularly in the fasting condition preceding TEE. Angiography and ICE yielded similar results when evaluating the LAA. Only 6.82% of patients in the ICE group and 9.85% in the TEE group had to switch to a different device size after the initial selection. Therefore, we conclude that the discrepancies in these measurements did not affect the device selection.

### 4.3 Procedural Complications and Follow-Up 

Despite the diverse and complex methods used in this combined procedure, the overall complication rate for 264 patients was only 3.79% in the TEE group and 4.54% in the ICE group, indicating comparable safety. Similar to the results by Alkhouli *et al*. [26], 3.3% and 4.1% of patients in the ICE and TEE groups experienced major procedure-related incidents, respectively (*p* = 0.76). Both groups had similarly low rates of all-cause death, stroke or transient ischemic attack, and severe bleeding; DRT and PDL were low and comparable in the two groups after a mean follow-up of 18.46 ± 7.70 months. The results of this study lend credence to concerns about the safety and effectiveness of the combined procedure.

Studies have indicated that left atrial ridge edema, which can occur after a combined ablation and LAAO procedure, can raise the postprocedural PDL rate. Li *et al*. [16] demonstrated that CA caused significant edema of the left atrial ridge, increasing the ridge from 4.6 ± 0.4 mm before CA to 6.8 ± 0.6 mm after CA (*p* < 0.01). At the 3-month follow-up in our study, the findings revealed similar rates of PDL between the TEE and ICE groups (23.81% vs. 24.62%). At the same time, the incidence of residual shunts measuring 3 to 5 mm was slightly reduced in the ICE group compared to the TEE group. However, the difference did not reach statistical significance (1.54% vs. 3.17%, *p* = 0.409). It is important to note that this lack of statistical significance may be attributed to the relatively small sample size in our study. No occurrences of severe PDL >5 mm were found in either group, demonstrating that the combined procedure does not exacerbate procedure-related PDL, which is a relatively common cause of procedure failure that can result in blood stasis and an increased risk of embolism. The rate of PDL after LAAO varied among different studies. Chen *et al*. [27] verified the PDL rate during the 52-day follow-up in a prospective cohort registration study. According to Park *et al*. [28], the incidence of PDL was 24.6%, 20.4%, and 41.7% at 1, 6, and 12 months, respectively. In a study by Wang *et al*. [29], the PDL rate was 17% after 3 months and steadily declined to 15.7% at 12 months after implantation. Prior research has linked PDL to an increased risk of LAA thrombosis and thromboembolic events [30]. However, a recent study revealed that a PDL of <5 mm is not associated with an increased risk of thromboembolism and does not necessitate further intervention [31].

## 5. Limitations 

This single-center study, encompassing a cohort of 264 patients, faces limitations in terms of generalizability. To substantiate the efficacy and safety of ICE guidance in combined procedures, it is imperative to conduct larger multicenter studies with extended follow-up periods. The restricted replicability of our findings is influenced by the involvement of a single, highly experienced staff member in our investigation.

## 6. Conclusions

This propensity score-matched study demonstrated that ICE guidance for a combined procedure exhibited comparable effectiveness and safety to TEE guidance. Moreover, ICE guidance offered the additional advantage of reducing both procedure time and fluoroscopy radiation dosage. Further research and increased clinical advocacy for ICE are essential to establish its standing as a viable alternative imaging modality.

## Data Availability

The datasets used or analyzed during the current study are available from the corresponding author upon reasonable request.

## Abbreviations

AF, atrial fibrillation; CA, catheter ablation; LAAO, left atrial appendage occlusion; TEE, transesophageal echocardiography; ICE, intracardiac echocardiography; LAA, left atrial appendage; PDL, peri-device leaks; DRT, device-related thrombus; TIAs/SE, transitory ischemic attacks/stroke episodes; SMD, standardized mean differences.

## Supplementary Material

Supplementary material associated with this article can be found, in the online version, at https://doi.org/10.31083/j.rcm2506192.
