# Supplementary figures and images for: Clinical Results and Safety of Intracardiac Echocardiography Guidance for Combined Catheter Ablation and Left Atrial Appendage Occlusion
Source: Rev Cardiovasc Med. 2024 May 27;25(6):192. doi: 10.31083/j.rcm2506192 (PMC11270113; doi:10.31083/j.rcm2506192)

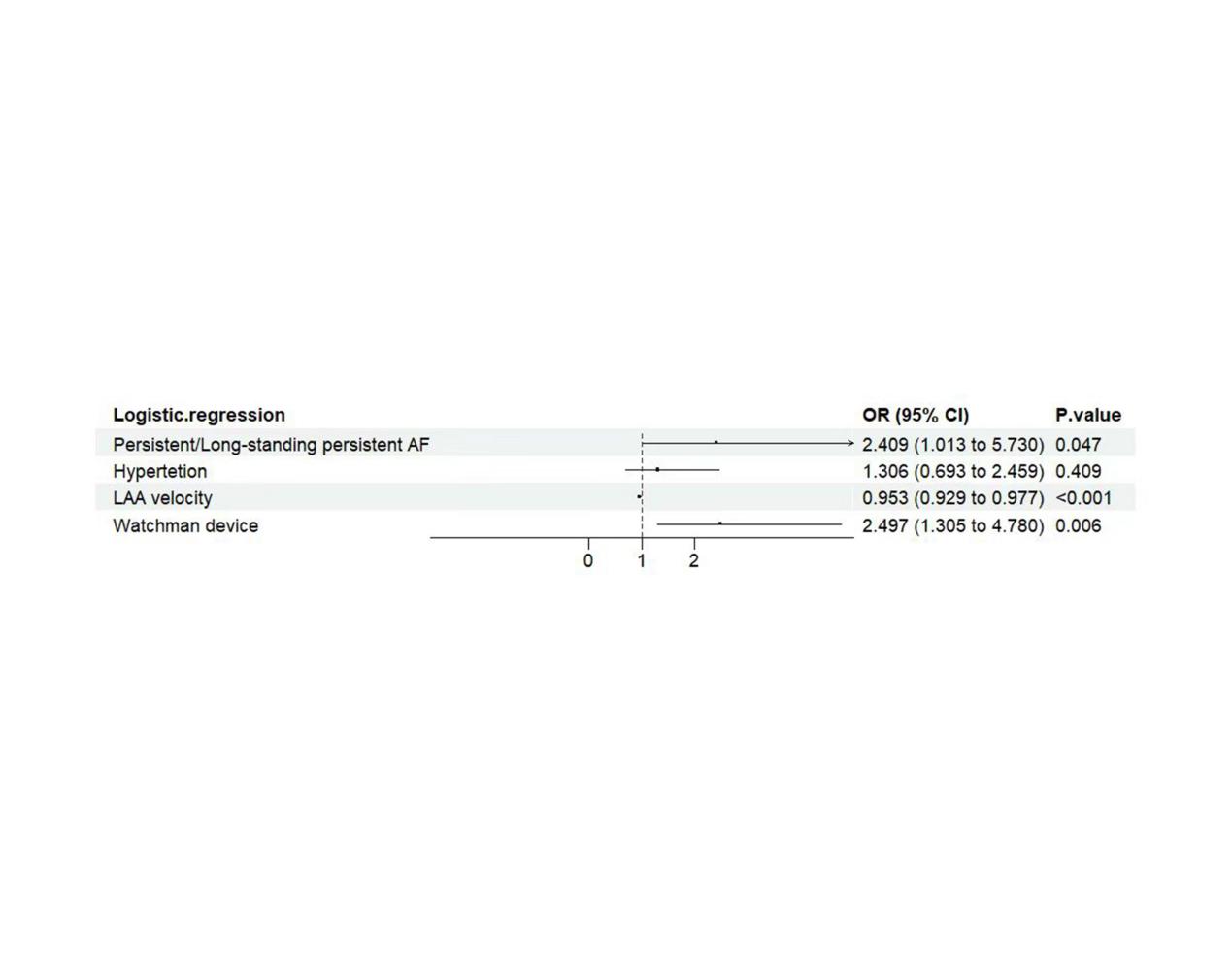


**Supplementary Fig. 1. Multivariate logistic regression analysis of the risk of PDL.**

Supplement: Supplementary file 1 [file 2153-8174-25-6-192-s1.docx]
